# A Comparison of Adaptive Behaviors among Mentally Retarded and Normal Individuals: A guide to Prevention and Treatment

**Published:** 2010

**Authors:** Leyla Sadrossadat, Alireza Moghaddami, Seyyed Jalal Sadrossadat

**Affiliations:** 1 Student of Clinical Psychology, Allient International University, California, USA; 2 PT, University of social welfare and Rehabilitation, Tehran, Iran; 3 Social Work, University of social welfare and Rehabilitation, Tehran, Iran

**Keywords:** Adaptive behaviors, Behavioral domains, Mental retardation, Prevention

## Abstract

**Objectives:**

Because of the importance of adaptive behaviors in social and domestic lives, this study aimed at a comparison of various domains of adaptive behaviors, between mentally retarded and normal individuals.

**Methods:**

A number of 246 normal and 74 mentally retarded individuals (7-18 years of age, mean: 12±3.5 years), participated this study in Tehran, Iran. Their adaptive behaviors scores, were obtained using “Adaptive Behavioral Scale, Residential & Community” (ABS-RC: 2), consisting of 18 domains of behavior. The scale was first translated into Persian by the professionals and then retranslated into English by another translator, to ensure content non-distortion.

**Results:**

The following domains were significantly lower in mentally retarded than in normal individuals: independent functioning, economic activity, language development, number & time, prevocational/vocational activity, self-direction, responsibility, socialization, disturbing interpersonal behavior, domestic activity, social engagement, conformity and trustworthiness. No significant difference was documented in the physical development, stereotype & hyperactive behaviors, sexual behavior as well as self abuse behavior domains, between the two groups.

**Conclusions:**

As mentally deficient subjects did worse than normal ones in terms of many adaptive behavioral domains, it implies that the adaptive behavioral issues in such people might need a great deal of attention and intervention. For these retarded people to function better in their social and residential environment, it would be necessary to develop their adaptive behaviors. This study may shed light on the importance of attention to the adaptive behavioral domains of mentally retarded people and also indicates the necessity of preventive measures, even for normal individuals.

## INTRODUCTION

As the diagnosis of mental deficiency was propounded, the relationship between this kind of disability with the adaptive behaviors was raised.^1^ Focusing on the concept of “adaptive behavior” was not the case, until the beginning of 1900s, during which, professionals just con-sidered low intellectual scores as the only crite-ria for diagnosing mental disability. In reality, intellectual scores and behavioral characteristics gradually changed into prominent criteria of mental disability, afterwards. Nonetheless, focusing on the “mere” intellectual test scores continued until 1930s, when Doll stated the social achievements as the leading criteria, to be worth receiving a great deal of attention in mental disability diagnosis area. Today, social achievement is an integral part of the behavior, and since 1959, the American Association of Mental Deficiency (AAMD), has minded “adaptive behavior” beside the intellectual scores, in its definition of mental deficiency.^2^ According to the definition that AAMD provides, adaptive behaviors and the degree to which these behaviors can influence mental deficiency are of great importance.^3^ Deficit in these behaviors means any failure to accomplish standards of independency and social tasks.^4^ Grossman defines adaptive behaviors as follows: “Fulfilling and/or the degree to which, one can perform social tasks, expected from the age and cultural class of the particular person”.^1^

Hunt and Marshall have noted that the adaptive behavior skills, such as personal and social competence, are weaker in the mentally deficient population, and these individuals, have difficulty in the adaptation to requirements of daily living.^5^ These behaviors include something more than just adaptation and coping with “out school environment” and they change according to the age and status of individuals.^6^ Looking at so called “requirements” and the trend, with which they change throughout the life, reveals dramatic and quick changes at infancy and preschool ages.^4^ An infant has to learn how to sit, stand and finally run. Moreover, the infant should after a while, be able to use spoon to eat and glass to drink. These examples show how expectations from an infant change fast, making the infant “adapt” behaviors to be capable of accommodating to the changing environment and age related requirements. Gradually, the child will learn to employ some kind of phonation for attracting significant others’ attention and shortly after, by repetition of single words as well as two word phrases, resulting in getting more deal of attention. At the preschool ages, the child acquires many fundamental skills, such as going to toilet, playing with age mates, and achieving interaction with older adults. During the school ages, the child has to “socialize” based upon the expectations at the school environment and respect some disciplines such as raising his/her hand to get permission, sitting still during the class sessions, and obeying teachers’ orders, for example. According to what AAMD notifies, social skills, like the above samples, are cornerstones of establishing successful communications with others, and also adapting self behaviors in accordance with what the “social environment” demands.^3^,^6^ In the second half of childhood ages as well as the initial years of adolescence, developing socialized behaviors, constitutes the mainest part of adaptive behaviors.^3^,^5^,^7^ These socialized behaviors and their level should always be assessed and considered when working with mentally retarded children and adolescents.^8^ Quantification and evaluation of adaptive behaviors are not as easy as quantifying “intelligence” which is calculated through some regular “intelligence quotient” (IQ) tests. This is to a major extent because adaptive behaviors are multidimensional traits and anyone who intends to evaluate and/or quantify them, must consider and obtain sufficient information concerning individual’s activity of daily living (ADL), activities at different social situations and also domestic relationships and behaviors.^7^

On the other hand, adaptive behaviors are among the indisputable factors of mental deficiency assessment and diagnosis.^1^ Therefore, in attempt to develop a quantification method for adaptive behaviors, it is inevitable to rely on direct inspection of individual’s above mentioned life dimensions, clinical judgment and utilization of some scales.^9^ Concluding the above discussed significances of the adaptive behaviors in mentally retarded population, this study was aimed at a comparison of adaptive behavior domains, in the mentally retarded subjects versus normal ones.

## METHODS

A number of 246 normal and 74 mentally retarded subjects whose ages ranged between 7 and 18 (12±3.5), were selected simply with no randomization, among those who volunteered for study participation (Table 1). These subjects were age and social class matched and their parents filled and signed written consent. Before taking into action for the main study procedure, the method of study and the instruction of filling scale items were explained in a convenient way, both to the parents and the study subjects. The second version of “Adaptive Behavior Scale-Residential & Community” (ABS-RC2), made and developed by the AAMD in 1993,^3^ was utilized to obtain scores of adaptive behavior domains, in the normal and mentally retarded participants. The scale is comprised of two major parts (related to domestic and social activities) and totally 18 domains of behavior. Initially, the scale was translated into Persian, and then, retranslated into English by two distinct qualified translators, to insure that the content of the scale is not distorted during the translation process. In the present study, both test-retest and internal consistency methods in order to calculate the internal consistency coefficients, as Nihara et al did before (1993).^3^

**Table 1:** Characteristics of the study participants

Gender	Normal	Retarded	Total
Female	125	38	157
Male	121	36	163
Total	246	74	320

Applying these methods, physical development domain, got the least coefficient (0.30) while social behavior domain, gained the highest coefficient (0.85). Reliability coefficients were derived from a test-retest method for each of the domains, in which, 30 examinees underwent two times of adaptive behavior test, with 8 weeks interval. Here, social behavior domain got the least coefficient value (0.66), while the highest value belonged to the social engagement domain (0.90).

After recruiting participants through a multistage sampling method, the scale sheets were filled out by the participants and/or the researchers, if some of the participants were not literated enough to fill the scales out by themselves. In the latter case, the researchers, questioned participants about the items of the scale to be answered, and then registered the participants’ answers.

### Statistical analysis:

Initially, mean values and standard deviations of adaptive behavior scores of normal and mentally deficient participants were calculated. Multivariable analysis of variance (M-ANOVA) test was conducted to reveal any possible significance of differences. A collection of tests, including Pillali-Bartlete’s trace, Wilk’s Lambda, Hotelling-Lawley’s trace, and Roy’s largest root test, were performed on the variables to estimate the effect of mental status on the adaptive behavior as dependent variable. Data was analyzed by using SPSS statistical package version 15.0 for windows (SPSS Inc., Chicago, USA). The significance level was set at p<0.05.

## RESULTS

The characteristics of participants are presented in Table 1. Mean values and standard deviations of adaptive behavior scores of normal and mentally deficient participants are presented in Table 2, and shows some differences between the two groups. As shown in Table 3, M-ANOVA reveals significant relationship between the subjects’ mental status and the general adaptive behavior (Pvalue=0.001). The value of Etta square (η^2^), 0.43, shows a moderate relationship between mental status and the total level of adaptive behavior. As the results, showed that there is a significant relationship between the two general variables (i.e., mental status and adaptive behavior), single variable ANOVA was needed to clarify the effects of subjects’ mental status on any of adaptive behavior domains. The results of the single variable ANOVA are summarized in Table 4. As in Table 4, calculated “F” for independent functioning, economic activity, language development, number and time, prevocational/vocational activity, selfdirection, responsibility, and disturbing interpersonal behavior were 19.16, 31.09, 61.90, 103.41, 11.36, 50.39, 53.02 and 12.68 respectively. Relevant P value of the above mentioned domains was 0.001. Therefore, mental status had significant effects on the above domains. Calculated F values for domains such as domestic activity, socialization, social behavior, consistency and trustworthiness were 5.32, 9.46, 5.62, 4.01 and 6.88, respectively, for which P value, exceeded 0.05 (alpha). Therefore, one cannot find any significant effects of the mental status of our subjects, on the mentioned behavioral domains. Normal subjects had better performances in the first set of behavioral domains than mentally retarded ones.

**Table 2:** Mean and standard deviation of adaptive behavior domains scores, in normal and mentally retarded individuals

Domains	Normal		Retarded	
	Mean	SD	Mean	SD
Independent Functioning	100.16	11.12	93.03	15.39
Physical Development	22.18	1.73	22.07	2.85
Economic Activity	15.81	4.12	12.68	4.48
Language Development	37.69	4.24	31.96	8.37
Numbers & Time	12.72	2.17	9.15	3.80
Domestic Activity	16.82	5.87	15.09	4.78
Vocational/Prevocational	8.82	0.5	7.89	2.28
Self Direction	19.71	3.67	15.86	5.18
Responsibility	8.99	1.42	7.41	2.19
Socialization	22.73	3.47	21.22	4.28
Social Behavior	7.56	8.83	10.36	8.85
Conformity	4.44	6.09	6.07	6.02
Trustworthiness	20.86	3.32	19.63	4.11
Stereotype Behaviors & Hyperactivity	4.59	6.39	5.59	6.94
Sexual Behavior	0.49	1.38	0.79	2.02
Self Abuse Behavior	10.54	1.31	10.73	1.76
Social engagement	2.32	3.56	2.15	3.99
Disturbing Interpersonal behavior	5.26	5.84	8.00	5.46

**Table 3:** M-ANOVA Results to show the effects of mental status (as an independent variable) on the adaptive behavior (as a dependent variable)

Variable	Test	Value	F	P	η^2^
Mental Status	Pillali- Bartlet’s	0.43	12.57	0.001	0.43
	Wilks’ Lambda	0.57	12.57	0.001	0.43
	Hotelling-Lawley’s	0.76	12.57	0.001	0.43
	Roy’s Largest root	0.76	12.57	0.001	0.43

**Table 4:** Single variable ANOVA results for any of the behavioral domains

Dependent Variables	Summation of Squares (SS)	Mean of Squares (MS)	Degree of Freedom (DF)	F	P
Independent Functioning	2863.88	2863.06	1	19.16	0.001
Physical Development	0.74	0.74	1	0.18	0.67
Economic Activity	550.05	550.05	1	31.09	0.001
Language Development	1847.17	1847.17	1	61.90	0.001
Numbers & Time	717.51	717.51	1	103.41	0.001
Domestic Activity	166.47	166.47	1	5.23	0.023
Vocational/Prevocational	49.07	49.07	1	11.36	0.001
Self Direction	834.2	834.2	1	50.39	0.001
Responsibility	140.56	140.56	1	53.02	0.001
Socialization	127.79	127.79	1	9.46	0.002
Social Behavior	438.70	438.70	1	5.62	0.018
Conformity	148.26	148.26	1	4.01	0.046
Trustworthiness	85.24	85.24	1	6.88	0.009
Stereotype Behaviors & Hyperactivity	55.47	55.47	1	1.31	0.254
Sexual Behavior	.5.22	5.22	1	2.17	0.141
Self Abuse Behavior	1.89	1.89	1	0.92	0.337
Social engagement	1.58	1.58	1	0.12	0.732
Disturbing Interpersonal behavior	420.61	420.61	1	12.68	0.001

## DISCUSSION

Results indicate that mentally retarded people performance, lacks significantly in many behavioral domains than the normal subjects. This failure to functioning is consistent with what Kopp, Baker and Brown found about the correlates of social skills in the preschoolers with developmental delays.^10^

Current research finding, match findings by some other researches, for example, what Jordan^11^ and Mac Millan^12^ described for language mal-development in mentally deficient people. Considering these facts, can be helpful in developing new therapy strategies.

There was no significant difference regarding physical development, stereo type behavior and hyperactivity, sexual behavior, self abuse behavior and social engagement between normal and mentally retarded subjects. This may indicate that some mentally retarded people have similar levels of abilities, in some of the behavioral domains, and this probably has been the case, for current research sample. Doing less in some adaptive behavior domains performance, can be result of etiology and type of the incident mental retardation, as mentally retarded people who have comorbidity of limb disorder, have to learn more movement skills and those who have comorbidity of family relationship disorders, need to acquire more social behavior skills. Besides, it is worth noting that mentally retarded sample of this research was of those who were “educable mentally deficient”, therefore, they are expected to have less behavioral disorders than severely retarded people. Normal children can also be defective in some of the above domains of behavior. So focusing on these domains, may be a good means by which several mal-adaptive behaviors can be prevented.
